# Bioleaching Microbial Community Metabolism and Composition Driven by Copper Sulphide Mineral Type

**DOI:** 10.1111/1758-2229.70261

**Published:** 2025-12-11

**Authors:** Katherine R. Lane, Sarah E. Jones, Thomas H. Osborne, David Geller‐McGrath, Bennet C. Nwaobi, LinXing Chen, Brian C. Thomas, Karen A. Hudson‐Edwards, Jillian F. Banfield, Joanne M. Santini

**Affiliations:** ^1^ Department of Earth and Planetary Sciences University of California Berkeley California USA; ^2^ Department of Civil and Environmental Engineering Massachusetts Institute of Technology Cambridge Massachusetts USA; ^3^ Institute of Structural and Molecular Biology, Division of Biosciences University College London UK; ^4^ Department of Earth, Atmospheric, and Planetary Sciences Massachusetts Institute of Technology Cambridge Massachusetts USA; ^5^ Camborne School of Mines and Environment and Sustainability Institute University of Exeter Penryn UK; ^6^ Department of Environmental Science, Policy and Management University of California Berkeley California USA

## Abstract

Copper bioleaching is a green technology for the recovery of copper from chalcopyrite (CuFeS_2_) and chalcocite (Cu_2_S) ores. Much remains to be learned about how mineral type and surface chemistry influence microbial community composition. Here, we established a microbial consortium from a copper bioleaching column in Cyprus on chalcopyrite and then sub‐cultured it to chalcocite to investigate how the community composition shifts due to changes in mineral structure and the absence of mineral‐derived Fe. The solution chemistry was determined and microbial communities characterised by genome‐resolved metagenomics after 4 and 8 weeks of cultivation. *Acidithiobacillus* species and strains, a *Rhodospirilales*, *Leptospirillum ferrodiazotrophum* and *Thermoplasmatales* archaea dominated all enrichments, and trends in abundance patterns were observed with mineralogy and surface‐attached versus planktonic conditions. Many bacteria had associated plasmids, some of which encoded metal resistance pathways, sulphur metabolic capacities and CRISPR‐Cas loci. CRISPR spacers on an *Acidithiobacillus* plasmid targeted plasmid‐borne conjugal transfer genes found in the same genus, likely belonging to another plasmid, evidence of intra‐plasmid competition. We conclude that the structure and composition of metal sulphide minerals select for distinct consortia and associated mobile elements, some of which have the potential to impact microbial activity during sulphide ore dissolution.

## Graphical abstract

Bioleaching is a microbially mediated green technology for the recovery of metals from ore. Here, microbial consortia from a copper bioleaching column in Cyprus were cultivated on chalcopyrite (CuFeS_2_) and then sub‐cultured on chalcocite (Cu_2_S) and chalcopyrite to investigate how mineral type and surface chemistry influence microbial community composition.

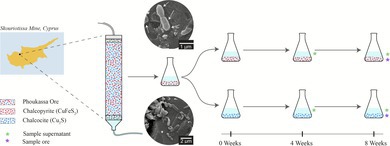

## 1 Introduction

Copper (Cu) has been used by humans since prehistoric times, and due to its malleability and high thermal and electrical conductivity, it is widely used in electronics, infrastructure, medical devices and renewable energy generation (Calvo and Valero 2022; Vera et al. 2022; Roberto and Schippers 2022). The vast majority of Cu in the Earth's crust occurs in sulphide minerals such as chalcocite (Cu_2_S), bornite (Cu_5_FeS_4_) and chalcopyrite (CuFeS_2_), with chalcopyrite accounting for 70% of crustal Cu and the majority of extracted Cu (Córdoba et al. 2008). The global demand for copper has significantly increased in recent decades; from 1991 to 2023, worldwide extraction more than doubled from 9.3 million to 22 million tons (Calvo et al. 2016; US Geological Survey 2024). Over time, Cu grade qualities have decreased as the easier‐to‐extract deposits have been sequentially exploited, thus causing the associated costs of mining, processing, transportation and extraction to rise. Therefore, industrial interest in bioleaching technologies for copper extraction as well as recycling (Baniasadi et al. 2021) has increased and today approximately 10%–20% of the world's copper production is extracted using these methods (Johnson and Roberto 2023). Bioleaching uses microorganisms to extract metals from ore, reducing associated economic costs and environmental impacts compared with traditional chemical leaching. Copper bioleaching involves applying dilute sulfuric acid over heaps or dumps of Cu‐bearing ores; iron‐ (Fe‐) and sulphur‐ (S‐)oxidising organisms mediate the bioleaching process, thus releasing copper sulphate salts into solution. These solutions are recycled back over the heaps along with the sulfuric acid for further enrichment of the copper. The Cu from the dilute sulphate solutions is then extracted, purified and enriched with the use of an organic solvent and recovered as metal ingots by electrolysis (electrowinning). Copper bioleaching is thought to be less harmful to the environment than smelting, as it produces fewer wind‐borne toxic elements and does not generate SO_2_(g), which can form acid rain (Nikolić et al. 2010; Vítková et al. 2011). However, the slower rate of chalcopyrite dissolution compared to other copper sulphides makes it difficult to bioleach (Yevenes 2009; Miki et al. 2011; Medina Ferrer et al. 2021). Therefore, improving methods to optimise the bioleaching of chalcopyrite is of economic interest to the Cu‐mining industry and of environmental interest to the wider society.

Acidophilic Fe‐ and S‐oxidising organisms from various phylogenetic groups have been identified in Cu‐bioleaching experiments (Zeng et al. 2010; Keeling et al. 2005; Mikkelsen et al. 2006; He et al. 2010; Chen et al. 2014). Bacteria from the genera *Leptospirillum*, *Acidithiobacillus* and *Sulfobacillus*; and Archaea from the genera *Ferroplasma* and *Sulfolobus* are the most common organisms found in chalcopyrite heap leaches and bioreactors (Zeng et al. 2010; Chen et al. 2014; Bakhti et al. 2024). The molecular mechanisms for S and Fe oxidation are distinct from each other, and some strains have been demonstrated to use only Fe (e.g., *Leptospirillum ferrooxidans* ) or S (e.g., *Acidithiobacillus thiooxidans* ) as sole electron donors, while others can use both (e.g., *Acidithiobacillus ferrooxidans* ) (Rawlings 2005). The capacity for N_2_ fixation by *Leptospirillum ferrodiazotrophum* was predicted from a metagenome‐derived genome (Tyson et al. 2004), and used to guide its isolation (Tyson et al. 2005). Subsequently, the ability to oxidise sulphur compounds was previously suggested; however, this has not been experimentally validated (Goltsman et al. 2009).

Previous investigations on the microbial populations responsible for Cu‐bioleaching from chalcopyrite‐containing ore used a mix of minerals (e.g., including pyrite) in the enrichment (Zeng et al. 2010; Keeling et al. 2005; Mikkelsen et al. 2006; He et al. 2010; Chen et al. 2014; Marhual et al. 2008; Zhou et al. 2009; Yu et al. 2014). While a mix of minerals is typical in heap leaches, these experiments do not distinguish between organisms that are using pyrite versus chalcopyrite, thus confounding the analysis of microbial‐mediated mechanisms. Furthermore, few studies have been performed with pure chalcopyrite, one of which was a monoculture of the Fe‐ and S‐oxidising bacterium, *A. ferrooxidans* (Zhao et al. 2013). Compared with chalcopyrite oxidation, chalcocite oxidation has been much less studied. Previous research demonstrated that oxidation of chalcocite by *Acidithiobacillus ferrooxidans* in sulfuric acid solutions at pH 1.7 resulted in the oxidation of the Cu^+^ in the chalcocite to Cu^2+^, and the subsequent formation of digenite (Cu_9_S_5_) and covellite (CuS) (Nielsen and Beck 1972).

**FIGURE 1:**
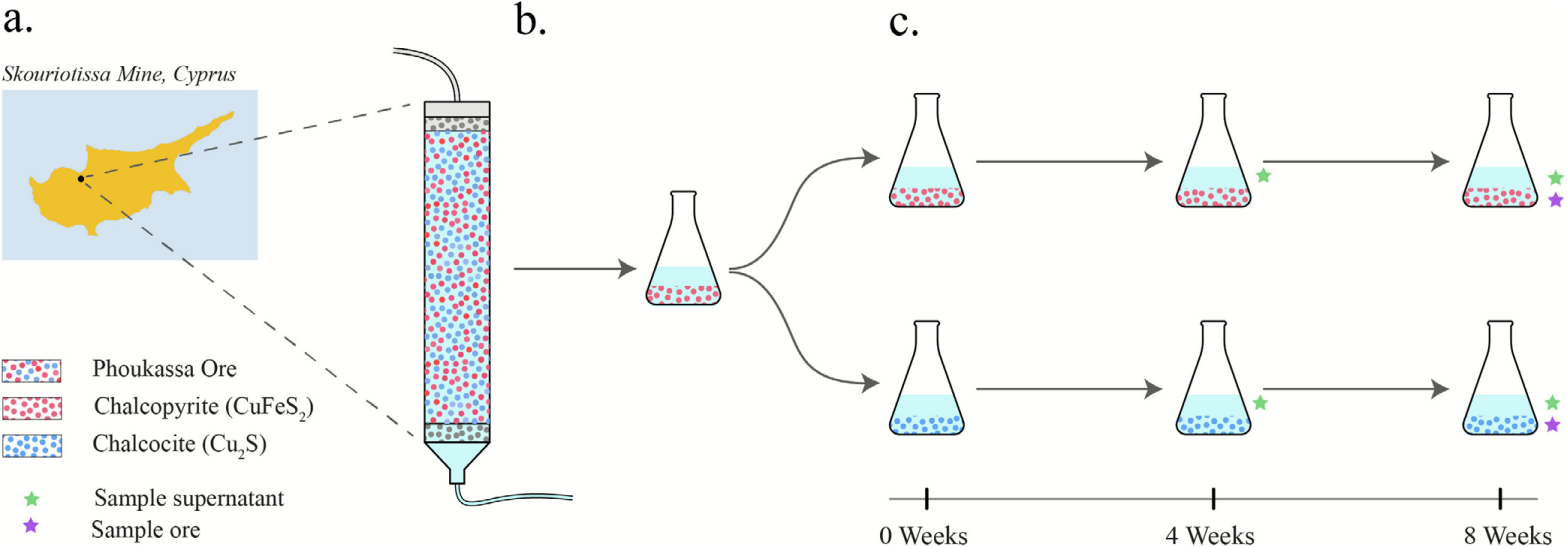
Schematic of metagenomic experimental design. The diagram illustrates the experimental design of the project. (a) Microbial consortium was sampled from the packed bioleaching column ‘SC3’ containing Phoukassa ore which has a natural mixture of chalcopyrite (red) and chalcocite (blue) at the Skouriotissa Mine in Cyprus. (b) The microbial community was cultivated and sub‐cultured in minimal acid medium with chalcopyrite 10 times. (c) Microbial community was sub‐cultured in minimal acid medium with chalcopyrite or chalcocite, and subsequently sub‐cultured on the same mineral five times prior to samples taken for DNA isolations. Metagenomes were sequenced from the supernatant at 4 weeks (green star), and from the supernatant (green star) and ore (blue star) separately at 8 weeks, totalling six metagenomes. Each metagenome consisted of DNA pooled from three replicates. Abiotic controls for each treatment consisted of minimal acid medium containing chalcopyrite or chalcocite (without the microbial inoculum) were sampled for chemical analysis at weeks 0, 4 and 6 to measure background abiotic dissolution processes.

Due to the presence or absence of iron in chalcopyrite and chalcocite, respectively, we hypothesised that these different mineral substrates would select for distinct microbial communities. In this study, we obtained a microbial consortium from a bioleaching column in Cyprus and enriched for Fe‐ and S‐oxidising organisms on chalcopyrite. We subsequently used this microbial enrichment to inoculate two experiments with (1) chalcocite and (2) chalcopyrite to investigate potential changes in the microbial communities (Figure 1). Microbial communities and their growth were characterised using chemistry, microscopy and metagenomics. We identify mobile elements with potential roles in microbial activity (e.g., metal resistance) and bioleaching capacity, define the importance of mineral type and report differences between planktonic and mineral surface‐attached consortia during microbially mediated dissolution of copper ore minerals.

## 2 Materials and Methods

### 2.1 Sample Collection and Enrichments

Microbial communities for the enrichment were sampled from the ‘SC3’ bioleaching column at the active Cu mine at Skouriotissa, Cyprus (35°5′28″ N 32°53′5″ E), which mines the Phoukassa orebody (Figure 1a). Skouriotissa is one of the oldest mines in Cyprus, operating since at least 2750 bc (Larnaca 1982). The deposit is a Cyprus‐type volcanogenic massive sulphide (Constantinou and Govett 1972) similar to others around the world mined for Cu. At Skouriotissa, bioleaching optimization experiments to improve Cu extraction were performed in columns with 2.8 wt.% Cu ore as substrate. A slurry of ore and microbes was sampled from leaching column ‘SC3’ operating at ambient temperature (16°C–26°C). The SC3 column was packed with local Phoukassa ore, in which the Cu‐bearing minerals were chalcopyrite, chalcocite, bornite and Cu‐bearing pyrite (FeS_2_) (unpublished data).

After sampling from the SC3 bioleaching column at the Skouriotissa mine in Cyprus, the SC3 Cu bioleaching consortium was enriched in a minimal acid medium (MAM pH 1.5) using pure chalcopyrite as the only mineral and energy source (Figure 1b). The microbial community was sub‐cultured into MAM pH 1.5 in two treatments: (1) with chalcocite as the mineral substrate and (2) with chalcopyrite to investigate the potential effects of these substrates on the microbial community (Figure 1c). Each treatment was accompanied by an abiotic control (three replicates), which included the minimal acid medium containing chalcocite or chalcopyrite, respectively, excluding the microbial community.

Approximately 0.5 g of slurry material from the SC3 bioleaching column was used to inoculate enrichments with minimal acid medium (MAM). Per litre, MAM contained: 0.4 g (NH_4_)_2_SO_4_, 0.4 g KH_2_PO_4_, 0.4 g MgSO_4_·7H_2_O, 1 mL, trace elements including W and Se (Atlas 2004), 0.25 g unsterilized research grade chalcopyrite (Sagewood Ltd.), and pH was adjusted to 1.5 with H_2_SO_4_. The minerals were not sterilised because doing so would affect the integrity and oxidation states; furthermore potential contamination of acidophiles from the surface of these minerals is unlikely, and no microbes were visualised on any of the abiotic controls. Research grade (i.e., pure mineral without impurities) chalcopyrite and chalcocite were ground to a maximum particle size of 50 μm and were characterised with X‐ray diffraction analysis (Phillips PW 1710 diffractometer using Cu‐Kα radiation and PANalytical X'Pert PRO diffractometer using Co‐Kα radiation). The purity of the minerals was confirmed by only peaks for chalcopyrite and chalcocite being present on the respective XRD spectra. Before the experiment, the microbial enrichment was incubated at 28°C without agitation and sub‐cultured 10 times with a 5% inoculum every 2 months in MAM with 0.25 g unsterilized research grade chalcopyrite (Figure 1b). The enrichment was then split and sub‐cultured with three replicates and three controls for each condition: MAM with 0.25 g unsterilized research grade chalcopyrite (Sagewood Ltd.), and MAM with 0.25 g unsterilized research grade chalcocite (Alfa Aesar) (Figure 1c). The control consisted of the MAM and chalcopyrite or chalcocite without the microbial consortia inoculum (microbes were not visualised on any of the abiotic controls).

### 2.2 Chemical Analyses

One mL samples from the 12 enrichments (two experimental conditions consisting of three replicates and three abiotic controls each) were collected at weeks 0, 4 and 8. Samples were centrifuged to remove cells and minerals, filtered (0.22 μm; Millipore), and stored at −20°C until analysis. Total soluble Cu, Fe and S concentrations were determined by inductively coupled plasma optical emission spectroscopy (ICP‐OES). Procedural blanks were run at the beginning and end of each analysis and at regular intermediate stages. The oxidation state of the soluble iron was determined using the colorimetric o‐Phenanthroline method (Saywell and Cunningham 1937).

### 2.3 Scanning Electron Microscopy

The SC3 consortium growing on chalcopyrite was imaged using a JEOL JSM‐6480LV high‐performance, variable pressure analytical scanning electron microscope (SEM) operating in low‐vacuum mode using 7–11 kV accelerating voltage and a spot size of 29 nm. Prior to examination, samples were mounted on 12.5 mm pin stubs with sticky carbon discs, freeze–dried in liquid nitrogen using a MODULO 4 k instrument for 30 min, and gold coated using a Polaron E5000 instrument.

### 2.4 DNA Extraction and Sequencing

Biotic enrichments with experimental conditions of 0.25 g chalcopyrite or chalcocite were sampled at 4 weeks (liquid phase) and 8 weeks (liquid phase and mineral‐attached), leading to a total of six samples for sequencing (Figure 1c). The supernatant and attached microbial communities at 8 weeks were sampled separately to compare the planktonic microbial community (‘supernatant’) and the community attached to the mineral surface (‘attached’). The supernatant was sampled using a pipette, taking care not to disturb the mineral and leaving about 1 mL of liquid remaining. The community on the mineral surface was sampled by washing with MAM. Samples were centrifuged to pellet cells. DNA was extracted from cell pellets using the MOBio PowerSoil DNA Isolation Kit (Qiagen, Germany), stored at −20°C and shipped on dry ice to RTL Genomics (Lubbock, TX, USA). Libraries were prepared at RTL Genomics (Lubbock, TX) with the KAPA HyperPrep Library Kit (KAPA Biosystems, Wilmington, MA), and samples were pooled equimolar according to the manufacturer's instructions. Libraries were sequenced on an Illumina HiSeq 2500 (Illumina, San Diego CA), producing 250 bp paired‐end reads. Raw reads are available on NCBI PRJNA 1170356, and related information is available in Table S1.

### 2.5 Metagenomic Assembly, Annotation and Binning

Raw read processing consisted of: removing Illumina adapters and contaminants with BBTools (Bushnell 2014), trimming reads with Sickle v1.33 (Joshi et al. 2011) and assessing quality before and after with FASTQC v0.11.5 (Andrews 2010), all with default parameters. Reads were assembled with IDBA‐UD v1.1.1 (IDBA‐UD 2022) with the following parameters ‘–mink 40–maxk 100–step 20’ and only assembled scaffolds of > 1000 bp in length were used in further analysis. Sequencing coverage of each scaffold > 1000 bp long was calculated by mapping raw reads against the assembly using Bowtie2 with default parameters (Langmead and Salzberg 2012). Prodigal v2.6.3 (Hyatt et al. 2010) was used with the ‘metagenomic’ setting to predict open reading frames (ORFs). ORF annotations were predicted with similarity searches using USEARCH ‘‐ublast’ (Edgar 2010) against UniProt Knowledgebase and UniRef100 databases (Suzek et al. 2007), and the Kyoto Encyclopaedia of Genes and Genomes (KEGG) (Ogata et al. 1999). Genes were also annotated with a custom set of HMMs using HMMER v3.3 (Johnson et al. 2010). tRNA sequences were predicted with tRNAscan‐SE (Schattner et al. 2005) and 16S rRNA sequences were predicted as previously described (Nawrocki et al. 2009) using ‘cmsearch’ from Infernal (Nawrocki et al. 2009) and SSU‐Align (Nawrocki 2009). Assembled scaffolds > 1000 bp in length and annotations were uploaded to ggKbase.

Genomic sequences for all organisms and plasmids were binned manually with the ggkbase interface using methods previously described (Brown et al. 2015; Anantharaman et al. 2016; Devoto et al. 2019) based on a combination of guanine‐cytosine content, DNA read coverage, taxonomic assignment and single copy gene content. Organisms were also binned automatically with ABAWACA (Brown et al. 2015) and metaBAT2 v2.12.1 (Kang et al. 2019). Of these manual and automated organism bins, the most complete and highest quality bins were selected from each sample with DASTool (Sieber et al. 2018) using default parameters. The resulting set of 56 total organism genomes from all samples was dereplicated using dRep v3.0.1 (Olm et al. 2017) at the 99% ANI threshold with the settings ‘–pa 0.5–sa 0.99’ yielding a set of 12 non‐redundant genomes. Similarly, the 72 total plasmid bins from all samples were dereplicated with the settings ‘–pa 0.5–sa 0.99–noQualityFiltering’ yielding 12 non‐redundant plasmids. Completeness and contamination of organism genomes was estimated with CheckM v1.1.3 (Parks et al. 2015) command ‘lineage_wf’ and default parameters. All genomes used in downstream analysis passed 70% completeness and 10% contamination thresholds. Furthermore, the 12 dereplicated genomes were classified with GTDB‐Tk v1.3.0 with ‘classify_wf’ using default parameters (Chaumeil et al. 2020). All non‐redundant organism and plasmid genomes are available at https://ggkbase.berkeley.edu/cu_bioleaching_organisms_and_plasmids and NCBI; Genbank Accession Numbers and associated information are listed in Table S2.

To explore plasmid replication, iRep script GC_skew.py (Brown et al. 2016) with default parameters was used to assess the GC skew and coverage of the 12 plasmids in the dereplicated set.

### 2.6 Metabolic Analysis

In addition to the above‐described annotation methods, Metabolic‐G.pl v2.0 was used with default parameters on the dereplicated set of 12 organism genomes to inform metabolic analysis in the system (Zhou et al. 2022).

### 2.7 Phylogenetic Analysis

The phylogenetic trees were constructed using sequences from the set of 12 non‐redundant genomes as well as sequences from acid mine drainage and analogous environments. Using GTOTree v1.6.12 (Lee 2019), bacterial and archaeal phylogenetic trees were constructed from concatenated single‐copy gene sets for bacteria (74 target genes) and archaea (76 target genes), respectively. By default, genomes with less than half of the targeted SCGs were excluded from downstream analysis. FastTree 2 v.2.1.10 was used to estimate the phylogenies, which were then midpoint rooted (Price et al. 2010).

### 2.8 CRISPR Analysis

To investigate the presence of CRISPR‐Cas systems encoded by contigs of bacterial, archaeal and plasmid contigs, we first searched their protein‐coding genes against the HMM databases of Cas proteins from TIGRFAM (Haft et al. 2003). For the contigs identified with at least one Cas protein, the upstream and downstream 10 kbp of the nucleotide sequences of the Cas protein(s) were searched for repeat array using PILER‐CR version 1.06 (Edgar 2007) with default parameters. The spacers from both the contigs, the mapped reads, and also the unplaced mapped reads were analysed as previously described (Chen et al. 2019). Blastn‐short was used to identify matches between CRISPR spacers and contigs from the sample, thus predicting the potential targets of the CRISPR spacers. Contigs that were hits of the spacers were filtered with alignment length > 24 bp and ≤ 1 mismatch as described previously (Al‐Shayeb et al. 2020). To further verify taxonomic assignment of hits from the spacer query, targeted proteins from the targeted contigs were searched against NCBI using blastp (BLAST 2022).

## 3 Results and Discussion

### 3.1 Cu Bioleaching by SC3 Microbial Consortium

**FIGURE 2:**
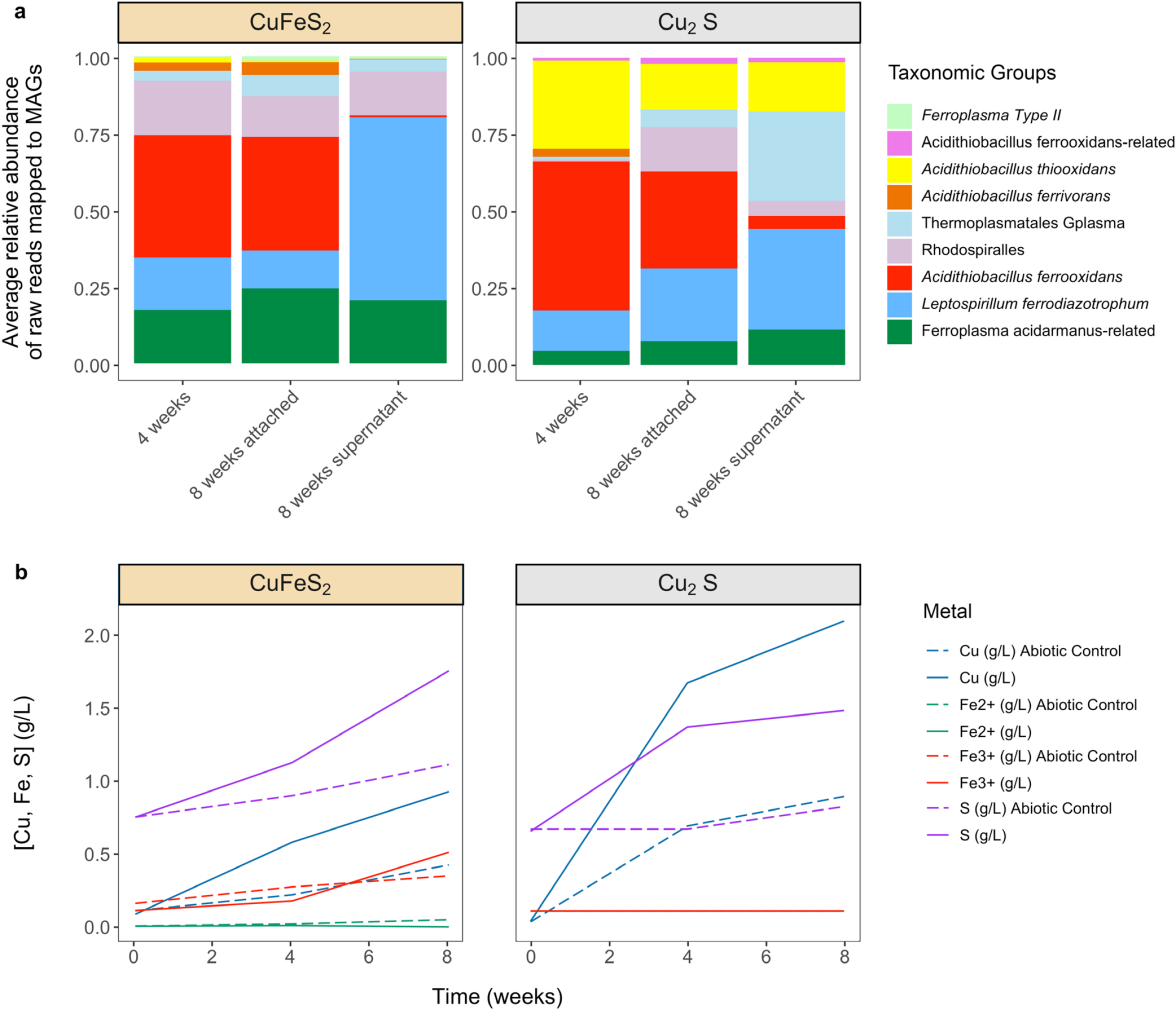
Community composition and chemical abundances over time with CuFeS_2_ and Cu_2_S cultivation. (a) Average relative abundance of raw reads mapped to MAGS of species groups (three replicates per timepoint). At Week 4, samples from the surface of mineral and fluid supernatant were mixed, while at Week 8 the supernatant and microbes attached to the surface of the metals were sampled separately. (b) Chemical abundances of abiotic controls (dashed lines) and treatment groups: CuFeS_2_ and Cu_2_S cultivation (solid lines).

Compared to the abiotic controls, the microbial consortia treatment groups with Cu‐containing minerals released 1.5–2.3 times more soluble Cu and S than the abiotic controls (Figure 2, Table S3). The soluble Fe concentration in the CuFeS_2_ cultures increased and was primarily Fe^3+^. However, overall the total amount of soluble Fe was lower than expected for stoichiometric dissolution of CuFeS_2_, possibly indicating the formation of iron‐containing minerals (Córdoba et al. 2008). The pH of the CuFeS_2_ cultures remained between 1.5 and 1.7 throughout the incubation, suggesting that any increase in pH from Fe oxidation was offset by the decrease in pH due to S oxidation to sulphate and the formation of iron‐containing minerals in the passivation layer such as jarosite (Córdoba et al. 2008; Blowes et al. 2003). The mechanism of chalcopyrite bioleaching via the polysulfide (H_2_S_*n*_) pathway is shown in the following equations (MS represents a metal sulphide, for example chalcopyrite) (Schippers and Sand 1999; Jones and Santini 2023; Baker and Banfield 2003):

(1) $$\mathrm{MS}+{\mathrm{Fe}}^{3+}+H^{+}\to {\mathrm{Cu}}^{2+}+0.5\;H_{2}S_{n}+{\mathrm{Fe}}^{2+}\;\left(n\geq 2\right)$$

(2) $${\mathrm{Fe}}^{2+}+0.25\;O_{2}+H^{+}\to {\mathrm{Fe}}^{3+}+0.5\;H_{2}O$$

(3) $$0.5\;H_{2}S_{n}+{\mathrm{Fe}}^{3+}\to 0.125\;S_{8}+{\mathrm{Fe}}^{2+}+H^{+}$$

(4) $$0.125\;S_{8}+1.5\;O_{2}+H_{2}O\to {{\mathrm{SO}}_{4}}^{2-}+{\text{2H}}^{+}$$

The pH of the Fe‐free Cu_2_S cultures (both abiotic and biotic) increased to 2.2–2.3, consistent with the oxidation of sulphide to elemental S without further oxidation of elemental S to sulphate (Miki et al. 2011). The equation for chalcocite oxidation is shown below (Fisher and Roman 1970):

(5) $${\mathrm{Cu}}_{2}S+0.5\;O_{2}+2\;H^{+}\to 2\;{\mathrm{Cu}}^{2+}+S^{0}+{\text{2H}}_{2}O$$

By 8 weeks, 2.26 times more Cu was bioleached in the chalcocite treatment (0.93 g/L) compared to the chalcopyrite (2.1 g/L). The structures of these minerals are distinct; in chalcopyrite, sulphur is in cubic closest packing with alternating trivalent iron and monovalent copper occupying half of the available tetrahedral sites whereas chalcocite contains sulphur arranged in hexagonal closest packing and only monovalent copper occupying all tetrahedral sites (Anthony et al. 2022). This difference in bioleaching efficiency could in part be due to the inorganic dissolution rates of chalcocite (faster) versus chalcopyrite (slower) under acidic conditions, as shown in previous experimental data (Yevenes 2009; Miki et al. 2011; Medina Ferrer et al. 2021; Neira et al. 2021).

### 3.2 Microbial Community Composition and Metabolism

Overall, the microbial communities cultivated on CuFeS_2_ and Cu_2_S included organisms typically found in bioleaching consortia: *Acidithiobacillus ferrooxidans* , *Acidithiobacillus thiooxidans* , *Acidithiobacillus ferrivorans* , *Leptospirillum ferrodiazotrophum*, *Ferroplasma acidarmanus*, Thermoplasmatales archaeon G‐plasma (also known as *Cuniculiplasma divulgatum*) and a Rhodospiralles species. At all time points, the most striking differences are the higher abundances of Rhodospiralles and the iron‐oxidising organisms *Ferroplasma acidarmanus* and *Leptospirillum ferrodiazotrophum* in the chalcopyrite enrichments and the higher abundance of sulphur‐oxidising *A. thiooxidans* and G‐plasma in the Cu_2_S enrichment (Figure 2a). Organisms are referred to in the manuscript by their long‐standing names for continuity with the literature (the current GTDB taxonomic assignment is detailed in Table S2). Archaeal and bacterial phylogenies are available in Figure S1.

After 4 weeks, both the CuFeS_2_ and Cu_2_S cultures were dominated by *A. ferrooxidans* . Both communities contained *L. ferrodiazotrophum*, reported to be an iron oxidizer (Tyson et al. 2005; Battaglia et al. 1994) supporting the inference that this bacterium can also grow on reduced sulphur compounds. The *L. ferrodiazotrophum* in this consortium contains genes for sulphur metabolism: sulphate adenylyltransferase, adenylylsulfate reductase and adenylylsulfate reductase subunit alpha (Table 1).

**TABLE 1:** Sulphur cycling‐related genes in dereplicated set of genomes.

Function	Gene	Gene name	AFe8S_6		Acidithiobacillus_ferrovorans‐related_58_37		Acidthiobacillus_ferrooxidans_59_545		Ferroplasma_acidarmanus_related_37_242		Leptospirillum_ferrodiazotrophum_59_239		Acidithiobacillales_thiooxidans_53_22		Rhodospirillales_66_495	
			Counts	Hits	Counts	Hits	Counts	Hits	Counts	Hits	Counts	Hits	Counts	Hits	Counts	Hits
Sulphide oxidation	sqr	Sulphide: quinone oxidoreductase	1	JSantini_Fe8S_scaffold_264_1	1	JSantini_Fe4_scaffold_217_9	1	JSantini_Fe4_scaffold_55_33	1	JSantini_Fe4_scaffold_137_25	1	JSantini_Fe4_scaffold_190_3	1	JSantini_Fe8_scaffold_12_67	1	JSantini_Fe8_scaffold_4_246
Sulphite reduction	dsrD	Dissimilatory sulphite reductase delta subunit	0	None	0	None	0	None	0	None	0	None	0	None	0	None
Sulphur oxidation	dsrA	Dissimilatory sulphite reductase alpha subunit	0	None	0	None	0	None	0	None	0	None	0	None	0	None
Sulphur oxidation	DsrB	Dissimilatory sulphite reductase beta subunit	0	None	0	None	0	None	0	None	0	None	0	None	0	None
Sulphur oxidation	sdo	Sulphur dioxygenase	0	None	2	JSantini_Fe4_scaffold_177_8,JSantini_Fe4_scaffold_484_3	3	JSantini_Fe4_scaffold_15_125,JSantini_Fe4_scaffold_292_16,JSantini_Fe4_scaffold_825_4	0	None	1	JSantini_Fe4_scaffold_99_5	2	JSantini_Fe8_scaffold_29_37,JSantini_Fe8_scaffold_30_40	1	JSantini_Fe8_scaffold_1_135
Sulphur reduction	sor	Sulphur oxygenase/reductase	0	None	1	JSantini_Fe4_scaffold_1007_4	0	None	1	JSantini_Fe4_scaffold_438_7	0	None	0	None	0	None
Sulphur reduction	sreA	Sulphur reductase molybdopterin subunit	0	None	0	None	0	None	0	None	0	None	0	None	0	None
Sulphur reduction	sreB	Sulphur reductase FeS subunit	0	None	0	None	0	None	0	None	0	None	0	None	0	None
Sulphur reduction	sreC	Sulphur reductase membrane anchor	0	None	0	None	0	None	0	None	0	None	0	None	0	None
Thiosulfate oxidation	soxB	S‐sulfosulfanyl‐l‐cysteine sulfohydrolase	0	None	1	JSantini_Fe4_scaffold_100_19	0	None	0	None	0	None	1	JSantini_Fe8_scaffold_80_24	0	None
Thiosulfate oxidation	soxY	Sulphur‐oxidising protein SoxY	0	None	2	JSantini_Fe4_scaffold_100_15,JSantini_Fe4_scaffold_668_5	0	None	0	None	0	None	2	JSantini_Fe8_scaffold_32_77,JSantini_Fe8_scaffold_80_28	0	None
Thiosulfate oxidation	soxC	Sulphane dehydrogenase subunit SoxC	0	None	0	None	0	None	0	None	0	None	0	None	0	None
Sulphite reduction	asrA	Anaerobic sulphite reductase subunit A	0	None	0	None	0	None	0	None	0	None	0	None	0	None
Sulphite reduction	asrB	Anaerobic sulphite reductase subunit B	0	None	0	None	0	None	0	None	0	None	0	None	0	None
Sulphite reduction	asrC	Anaerobic sulphite reductase subunit C	0	None	0	None	0	None	0	None	0	None	0	None	0	None
Sulphate reduction	aprA	adenylylsulfate reductase, subunit A	0	None	0	None	0	None	0	None	0	JSantini_Fe4_scaffold_129_25	0	None	0	None
Sulphate reduction	Sat	Sulphate adenylyltransferase	1	JSantini_Fe8S_scaffold_787_4	1	JSantini_Fe4_scaffold_561_2	1	JSantini_Fe4_scaffold_12_41	0	None	1	JSantini_Fe4_scaffold_129_23	2	JSantini_Fe8_scaffold_32_65,JSantini_Fe8_scaffold_3533_2	0	None
Sulphate reduction	cysC	Adenylylsulfate kinase	0	None	1	JSantini_Fe4_scaffold_561_2	1	JSantini_Fe4_scaffold_12_41	0	None	0	JSantini_Fe4_scaffold_129_24	1	JSantini_Fe8_scaffold_32_65	1	JSantini_Fe8_scaffold_15_12
Sulphate reduction	cysN	Bifunctional enzyme CysN/CysC	0	None	0	None	1	JSantini_Fe4_scaffold_230_8	0	None	0	None	0	None	0	None
Thiosulfate disproportionation	phsA	Thiosulfate reductase/polysulfide reductase chain A	0	None	0	None	0	None	0	None	0	None	0	None	0	None

Function	Gene	Gene name	Acidithiobacillus_ferrivorans_57_58		Ferroplasma_acidarmanus_36_84		Gplasma_37_19		Acidithiobacillus_ferrooxidans‐related_58_37		Acidithiobacillus_thiooxidans_53_1337		
			Counts	Hits	Counts	Hits	Counts	Hits	Counts	Hits	Counts	Hits	Hmm detecting threshold
Sulphide oxidation	sqr	Sulphide: quinone oxidoreductase	1	JSantini_S4_scaffold_11_52	1	JSantini_S4_scaffold_93_25	1	JSantini_S4_scaffold_1240_3	1	JSantini_S8_scaffold_2232_1	1	JSantini_SS8_scaffold_73_23	300|full
Sulphite reduction	dsrD	Dissimilatory sulphite reductase delta subunit	0	None	0	None	0	None	0	None	0	None	32.80|full
Sulphur oxidation	dsrA	Dissimilatory sulphite reductase alpha subunit	0	None	0	None	0	None	0	None	0	None	200.00|full
Sulphur oxidation	DsrB	Dissimilatory sulphite reductase beta subunit	0	None	0	None	0	None	0	None	0	None	204.00|full
Sulphur oxidation	sdo	Sulphur dioxygenase	4	JSantini_S4_scaffold_139_18,JSantini_S4_scaffold_2_93,JSantini_S4_scaffold_1961_3,JSantini_S4_scaffold_11_69	0	None	1	JSantini_S4_scaffold_1833_5	5	JSantini_S8_scaffold_443_4,JSantini_S8_scaffold_1319_3,JSantini_S8_scaffold_1820_3,JSantini_S8_scaffold_238_13,JSantini_S8_scaffold_1654_2	2	JSantini_SS8_scaffold_138_5,JSantini_SS8_scaffold_2601_2	120|full
Sulphur reduction	sor	Sulphur oxygenase/reductase	1	JSantini_S4_scaffold_49_2	0	None	0	None	0	None	0	None	300|full
Sulphur reduction	sreA	Sulphur reductase molybdopterin subunit	0	None	0	None	0	None	0	None	0	None	2530.13|full
Sulphur reduction	sreB	Sulphur reductase FeS subunit	0	None	0	None	0	None	0	None	0	None	545.67|full
Sulphur reduction	sreC	Sulphur reductase membrane anchor	0	None	0	None	0	None	0	None	0	None	987.53|full
Thiosulfate oxidation	soxB	S‐sulfosulfanyl‐l‐cysteine sulfohydrolase	1	JSantini_S4_scaffold_3_91	0	None	0	None	0	None	0	None	550.00|full
Thiosulfate oxidation	soxY	Sulphur‐oxidising protein SoxY	2	JSantini_S4_scaffold_3_88,JSantini_S4_scaffold_2_26	0	None	0	None	0	None	2	JSantini_SS8_scaffold_693_4,JSantini_SS8_scaffold_207_30	125.00|full
Thiosulfate oxidation	soxC	Sulphane dehydrogenase subunit SoxC	0	None	0	None	0	None	0	None	0	None	320.00|full
Sulphite reduction	asrA	Anaerobic sulphite reductase subunit A	0	None	0	None	0	None	0	None	0	None	323.15|full
Sulphite reduction	asrB	Anaerobic sulphite reductase subunit B	0	None	0	None	0	None	0	None	0	None	288.60|full
Sulphite reduction	asrC	Anaerobic sulphite reductase subunit C	0	None	0	None	0	None	0	None	0	None	324.10|full
Sulphate reduction	aprA	adenylylsulfate reductase, subunit A	0	None	0	None	0	None	0	None	0	None	641.75|full
Sulphate reduction	Sat	Sulphate adenylyltransferase	0	None	0	None	0	None	2	JSantini_S8_scaffold_528_8,JSantini_S8_scaffold_4433_2	0	None	181.80|full
Sulphate reduction	cysC	Adenylylsulfate kinase	0	None	0	None	0	None	2	JSantini_S8_scaffold_297_7,JSantini_S8_scaffold_1069_6	1	JSantini_SS8_scaffold_1315_2	133.35|full
Sulphate reduction	cysN	Bifunctional enzyme CysN/CysC	0	None	0	None	0	None	2	JSantini_S8_scaffold_297_7,JSantini_S8_scaffold_186_18	0	None	327.65|full
Thiosulfate disproportionation	phsA	Thiosulfate reductase/polysulfide reductase chain A	0	None	0	None	0	None	0	None	0	None	323|full

**FIGURE 3:**
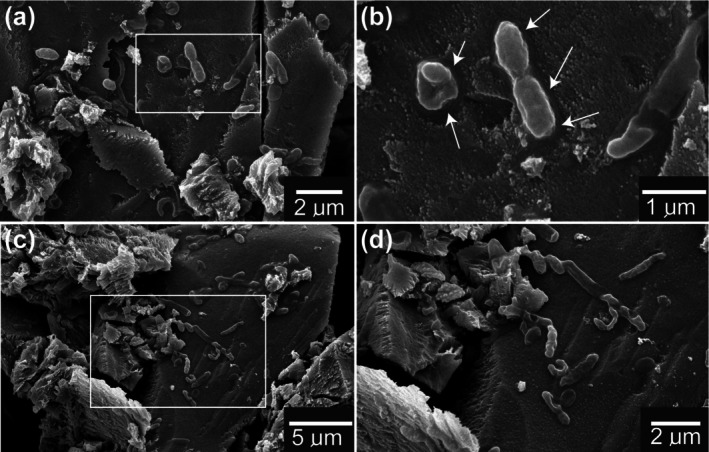
Scanning electron micrograph images of SC3 consortium on CuFeS_2_. Different sub‐samples of the mineral are shown in (a) and (c) with a magnified image shown in (b) and (d), respectively. Arrows point to small dissolution pits or the removal of a surface coating underneath the microbial cells.

The composition of the chalcocite versus chalcopyrite communities and mineral‐attached versus planktonic communities was distinct (Figure 2a). At 8 weeks, the CuFeS_2_ surface‐attached community was similar to the overall 4‐week community, whereas the planktonic fraction had a high abundance of *L. ferrodiazotrophum* and a low abundance of *A. ferrooxidans* . These findings suggest that *L. ferrodiazotrophum* oxidises dissolved intermediate sulphur compounds (e.g., thiosulfate/tetrathionate/sulphite) whereas *A. ferrooxidans* may contribute to the oxidation of surface‐bound compounds such as polysulfide. Furthermore, SEM images of the CuFeS_2_ community on the surface of chalcopyrite showed microbes with varied morphologies (Figure 3) and the observed mineral surface may be a surface coating that has been removed under the cells. Small dissolution pits may have been formed biotically or abiotically, consistent with what has been found previously for bioleaching of sulphide minerals (Edwards et al. 2001).

Compared to the Cu_2_S 4‐week community, the 8‐week surface‐attached community had a greater relative abundance of *L. ferrodiazotrophum* and Rhodospiralles. G‐plasma is more abundant in the planktonic fraction from the Cu_2_S enrichments, consistent with its growth primarily via the oxidation of dissolved intermediate sulphur compounds, as it contains the two genes *sqr* (sulphide:quinone oxidoreductase) and *sdo* (sulphur dioxygenase) (Table 1). Previously, a G‐plasma genome was reported that contained a rhodanese‐like domain on a genomic island, indicating that it was likely acquired by horizontal gene transfer (Yelton et al. 2013).

One of the most striking findings is the low abundance of *A. ferrooxidans* in the supernatant regardless of the mineral type used in the enrichment. This result points to a preference for the oxidation of surface‐bound sulphur compounds over dissolved Fe^2+^, which is regenerated in chalcopyrite‐based experiments following its reaction of Fe^3+^ with reduced sulphur at mineral surfaces.

In contrast to the abundance of *A. ferrooxidans* , *A. thiooxidans* (sulphur oxidizer) is present in similar abundances in both the surface and supernatant of the CuS_2_ enrichment and low in the CuFeS_2_ treatment. This suggests that in the absence of iron, *A. thiooxidans* may be a more competitive sulphur oxidizer at both the surface and in the supernatant.

Overall, *A. ferrivorans* has a similar low abundance at 4 weeks in CuFeS_2_ and CuS_2_, and at 8 weeks attached in CuFeS_2_ but negligible abundance at 8 weeks in the CuFeS_2_ supernatant, and 8 weeks attached and supernatant in CuS_2_. Similar to *A. ferroxidans* in being a sulphur and iron oxidizer, *A. ferrivorans* ’ abundance distribution is distinct. Differences may be explained in variations in their iron oxidation pathways and/or regulation of these pathways (Hallberg et al. 2009). Furthermore, each microbial community has distinct functional and ecological partitioning, and the ecological community dynamics of this inoculum may explain these differences in distribution.

Our results underline the partitioning of functions in the consortia, with iron oxidation likely carried out by planktonic *L. ferrodiazotrophum*. The produced ferric iron drives the oxidation of mineral‐associated reduced sulphur, contributing to the removal of intermediate sulphur compounds from mineral surfaces. A combination of other bacteria and archaea likely completes the sulphur oxidation pathway in solution (e.g., with primarily *Ferroplasma*, Rhodospiralles, and possibly *L. ferrodiazotrophum* in chalcopyrite enrichments and *G‐plasma*, *L. ferrodiazotrophum* and *A. thiooxidans* in chalcocite experiments). As *A. thiooxidans* is known to oxidise elemental sulphur (Tyson et al. 2005; Suzuki et al. 1992; Lara et al. 2013), the overall enrichment of *A. thiooxidans* in the Cu_2_S cultures could reflect the development of appreciable elemental sulphur only on the surface of chalcocite (Kitai et al. 2022).

**FIGURE 4:**
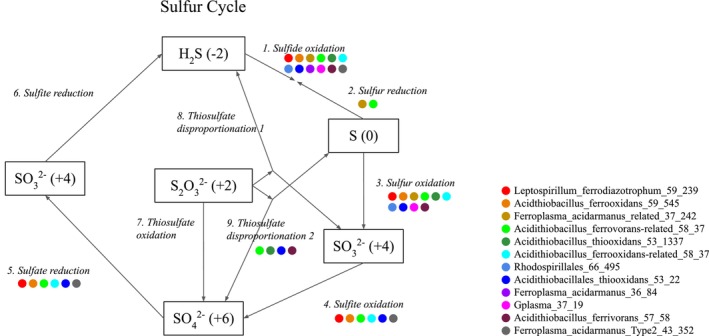
Sulphur cycling of SC3 microbial consortia. The coloured circles represent organisms containing the genes for these transformations.

**FIGURE 5:**
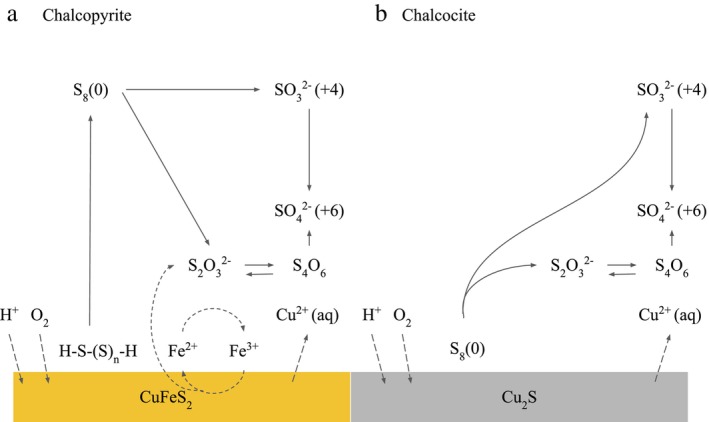
Proposed bioleaching mechanisms of chalcopyrite (A) and chalcocite (B) SC3 bioleaching microbial consortia based on the literature (Schippers and Sand 1999; Jones and Santini 2023; Baker and Banfield 2003; Lara et al. 2013).

Overall sulphur metabolisms predicted from the dereplicated set of genomes are shown in Figure 4. These metabolisms show genetic capabilities from the metagenome‐assembled genomes, not activity of these organisms in the enrichments. Figure 5 illustrates the proposed bioleaching mechanisms of the consortia on chalcopyrite (5A) and chalcocite (5B) (Schippers and Sand 1999; Jones and Santini 2023; Baker and Banfield 2003; Lara et al. 2013).

### 3.3 Mobile Elements in SC3 Bioleaching Community

The role of mobile elements such as plasmids and prokaryotic viruses (phages) in transferring genes involved in metabolic processes and resistance to stress such as antibiotics is well known (Palomino et al. 2023; Acman et al. 2020). Moreover, phages carrying auxiliary metabolic genes (AMGs) have been shown to play critical roles in photosynthesis (Mann et al. 2003), the metabolism of sulphur in microbiomes from diverse ecosystems (Kieft et al. 2021), and in the aerobic oxidation of methane in freshwater (Kieft et al. 2021). Mobile elements may also play important roles in microbial ecology and evolution; we identified 12 distinct plasmids, some of which carry genes involved in metal resistance, metabolism and CRISPR‐Cas loci (Table 2). The 128 kbp Plasmid_64_937 with genes phylogenetically most similar to those of Alphaproteobacteria contained two putative copper resistance genes. *Acidithiobacillus* Plasmid_56_860 encodes a putative sulphate adenylyltransferase, which plays a key role in assimilatory sulphur reduction and dissimilatory sulphur oxidation and reduction. Plasmid_55_30 also encodes a putative rusticyanin, an essential component of the Fe(II) oxidation electron transport chain. Plasmid_59_32 encodes a putative nitrite reductase. GC skews were low and the patterns were noisy, but three of the plasmids' patterns are suggestive of rolling circle replication (Figure S2).

**TABLE 2:** Functional genes contained in dereplicated set of plasmid genomes.

Plasmid	Zinc‐containing alcohol dehydrogenase	Pili_PilT_Twitching_NTPase	Pili_pilB_traffic_NTPase	Pili: PilD peptidase A24A domain‐containing protein	Pili_Pilins	Flagella_related proteins	Transketolase	Adenylosuccinate lyase	Cytochrome_c_Biogenesis	ABC transporter‐related protein	Sulphate adenylyltransferase
Plasmid_Leptospirillum_56_137	0	0	0	0	0	JSantini_SS8_scaffold_33_54, JSantini_SS8_scaffold_33_55, JSantini_SS8_scaffold_33_66	0	JSantini_SS8_scaffold_33_83	0	0	0
Plasmid_Acidithiobacillus_59_32	0	0	0	0	0	0	0	0	0	0	0
Plasmid_Alphaproteobacteria_64_937	0	0	0	0	0	0	JSantini_Fe8S_scaffold_17_22, JSantini_Fe8S_scaffold_17_23	0	JSantini_Fe8S_scaffold_17_97, JSantini_Fe8S_scaffold_17_101	JSantini_Fe8S_scaffold_17_24, JSantini_Fe8S_scaffold_17_25, JSantini_Fe8S_scaffold_17_26, JSantini_Fe8S_scaffold_17_33	0
Plasmid_Acidithiobacillus_50_48	0	0	0	0	0	0	0	0	0	0	0
Plasmid_Acidithiobacillus_56_860	0	JSantini_S8_scaffold_14_21	JSantini_S8_scaffold_14_114	JSantini_S8_scaffold_14_116	0	0	0	0	0	0	JSantini_S8_scaffold_14_102
Plasmid_57_162	0	0	0	0	JSantini_S8_scaffold_17_88	0	0	0	0	0	0
Plasmid_Acidithiobacillus_56_509	0	0	0	0	0	0	0	0	0	0	0
Plasmid_Acidithiobacillus_58_640	0	0	0	0	JSantini_SS8_scaffold_35_97	0	0	0	0	0	0
Plasmid_Acidithiobacillus_60_86	0	JSantini_Fe8_scaffold_50_40, JSantini_Fe8_scaffold_50_60	0	0	0	0	0	0	0	0	0
Plasmid_Acidithiobacillus_55_30	0	0	0	0	0	0	0	0	0	0	0
Plasmid_51_263	JSantini_S8_scaffold_27_10	0	0	0	0	0	0	0	0	0	0
Plasmid_53_253	0	0	0	0	0	0	0	0	0	0	0

Plasmid	Molybdopterin oxidoreductase	Nitrite reductase (NAD(P)H) small subunit	Nitrite reductase (NAD(P)H) large subunit	Amino acid biosynthesis: dihydroxy‐acid dehydratase	Adenylosuccinate_lyase	Serine peptidase: peptidase S26	Putative copper resistance protein	CRISPR associated	CRISPR locus	Rusticyanin		
Plasmid_Leptospirillum_56_137	0	0	0	0	JSantini_SS8_scaffold_33_83	0	0	0	0	0		
Plasmid_Acidithiobacillus_59_32	JSantini_Fe8S_scaffold_42_41	JSantini_Fe8S_scaffold_42_42	JSantini_Fe8S_scaffold_42_43	0	0	0	0	0	0	0		
Plasmid_Alphaproteobacteria_64_937	0	0	0	0	0	0	JSantini_Fe8S_scaffold_17_93, JSantini_Fe8S_scaffold_17_94	0	0	0		
Plasmid_Acidithiobacillus_50_48	0	0	0	0	0	0	0	0	0	0		
Plasmid_Acidithiobacillus_56_860	0	0	0	0	0	0	0	0	0	0		
Plasmid_57_162	0	0	0	0	0	JSantini_S8_scaffold_17_73	0	0	0	0		
Plasmid_Acidithiobacillus_56_509	0	0	0	0	0	0	0	JSantini_SS8_scaffold_20_100	JSantini_SS8_scaffold_20	0		
Plasmid_Acidithiobacillus_58_640	0	0	0	0	0	0	0	0	0	0		
Plasmid_Acidithiobacillus_60_86	0	0	0	0	0	0	0	JSantini_Fe8_scaffold_50_79		0		
Plasmid_Acidithiobacillus_55_30	0	0	0	0	0	0	0	0	0	JSantini_Fe8S_scaffold_40_45		
Plasmid_51_263	0	0	0	JSantini_S8_scaffold_27_1	0	0	0	0	0	0		
Plasmid_53_253	0	0	0	0	0	0	0	0	0	0		

Two plasmids contained CRISPR‐associated genes, and Plasmid_56_509, phylogenetically linked to *Acidithiobacillus*, had an associated CRISPR locus. CRISPR spacers on this plasmid targeted two unbinned contigs taxonomically categorised as *Acidithiobacillus* in the same sample (Table 3). Both contigs encoded genes annotated as ‘conjugal transfer proteins or related’ and other plasmid‐related genes such as Type IV secretion systems. Thus, Plasmid_56_509 is inferred to use a CRISPR‐Cas system to target plasmids of the same genus, that is, evidence of intra‐plasmid competition. This has previously been reported for plasmids of *Leptospirillum* that target other *Leptospirillum* plasmids (Goltsman et al. 2009) and it has been shown that CRISPR‐Cas systems often target other plasmids associated with the same species (Pinilla‐Redondo et al. 2022). As shown in this bioleaching consortia, plasmid–plasmid competition dynamics may be more prevalent than previously recognised. The inventory of plasmids reported here expands the database of plasmids from acidophiles. Considering the rapidly expanding research on extrachromosomal elements (Yu et al. 2024; Al‐Shayeb et al. 2022; Zheludev et al. 2024) and engineering of microbial consortia (Rubin et al. 2022; Wang et al. 2023; Ronda et al. 2019), these mobile elements may find application in future experiments that adapt these genetic elements for delivery of genome editing tools into microbes within consortia, possibly to improve bioleaching performance. With the decrease in ore grade and high demand for rare earth elements for the green energy transition, researchers are currently using directed evolution and engineering individual microbes to increase yield and expand applications (Schmitz et al. 2025; Jung et al. 2023); targeted engineering of consortia with genetic elements may enable a systems approach to fill this gap.

**TABLE 3:** Plasmid‐associated CRISPR loci.

Plasmid	Scaffold/gene	Annotation									
(A) Plasmids in dereplicated set with CRISPR‐associated genes											
Plasmid_Acidithiobacillus_56_509	JSantini_SS8_scaffold_20_100	CRISPR type III‐associated RAMP protein									
Plasmid_Acidithiobacillus_60_86	JSantini_Fe8_scaffold_50_79	CRISPR‐associated RAMP Cmr4 family protein family protein									

Scaffold	Bin	Taxonomy	# spacers	Scaffold length (bp)	Scaffold GC%	Scaffold coverage	Neighbouring genes				
(B) Scaffolds targeted by CRISPR spacers from Plasmid_56_509, Scaffold/gene: JSantini_SS8_scaffold_20_100											
JSantini_SS8_scaffold_31	JSantini_SS8_UNK	NCBI: *Acidithiobacillus ferrivorans*	4	80,146	56.65	96.63	Conjugal transfer protein TrbG/VirB9/CagX, conjugal transfer protein, P‐type conjugative transfer protein TrbL, P‐type conjugative transfer protein TrbJ, CagE, TrbE, VirB component of type IV transporter system, conjugal transfer protein TrbC, P‐type conjugative transfer ATPase TrbB, P‐type conjugative transfer ATPase TrbB, DNA topoisomerase III, TRAG family protein, Uncharacterized protein *n* = 1 Tax = mine drainage metagenome RepID = E6QCA8_9ZZZZ (and many more ‐ this is a large contig)				
JSantini_SS8_scaffold_4334	JSantini_SS8_UNK	NCBI: Acidithiobacillus ferrianus/ferrooxidans	3	1134	52.56	3.53	trwC; putative relaxase TrwC, Putative plasmid transfer protein TraC				

Query	Subject or target	Pident (percentage of identical matches)	Length	# mismatch	# gap openings	Qstart (start of alignment in query)	Qend	Sstart (start of alignment in subject)	Send	Evalue	Bitscore
(C) Spacer matches to JSantini_SS8_scaffold_31 and JSantini_SS8_scaffold_4334											
JSantini_SS8_scaffold_31	JSantini_SS8_scaffold_20_region_63235_64908_spacer_1028	96	25	1	0	17,550	17,574	7	31	2.94E‐04	42.1
JSantini_SS8_scaffold_31	JSantini_SS8_scaffold_20_region_63235_64908_spacer_1026	96	25	1	0	17,550	17,574	7	31	2.94E‐04	42.1
JSantini_SS8_scaffold_31	JSantini_SS8_scaffold_20_region_63235_64908_spacer_1025	96	25	1	0	17,550	17,574	7	31	2.94E‐04	42.1
JSantini_SS8_scaffold_31	JSantini_SS8_scaffold_20_region_63235_64908_spacer_985	96	25	1	0	17,550	17,574	7	31	2.94E‐04	42.1
JSantini_SS8_scaffold_4334	JSantini_SS8_scaffold_20_region_63235_64908_spacer_845	96.296	27	1	0	960	986	31	5	3.11E‐07	46.1
JSantini_SS8_scaffold_4334	JSantini_SS8_scaffold_20_region_63235_64908_spacer_888	96.552	29	1	0	962	990	29	1	1.99E‐08	50.1
JSantini_SS8_scaffold_4334	JSantini_SS8_scaffold_20_region_63235_64908_spacer_17	96.774	31	1	0	960	990	31	1	1.27E‐09	54

## 4 Conclusions

The SC3 bioleaching microbial consortia were characterised using chemical analyses, SEM microscopy and genomics. This investigation demonstrates the importance of combining single mineral bioleaching experiments with metagenomics. Here, we show how mineral type drives microbial community composition and metabolism in both planktonic and mineral‐attached consortia during microbially mediated dissolution of copper ore minerals. Our results provide new insights into how the availability of different sulphur compounds shapes the bioleaching microbial community and the roles of plasmids in these systems. Importantly, the data constrain the capacities of specific organisms, such as oxidation of intermediate sulphur compounds, that can only be partially predicted based on gene content.

A frontier in microbiology research is the use of genome editing tools to modify microbes without removing the organism from the community, thus preserving key microbe–microbe interactions (Rubin et al. 2022). Two requirements for such experiments are (i) the availability of realistic, stable laboratory consortia in which to perform experiments and (ii) mechanisms to effectively deliver editing tools to specific consortia members with high efficiency. Given the results of this study, we suggest that future work might leverage enrichments and plasmids such as reported here to perform such experiments. This approach could elucidate how these communities function as the result of individual and interconnected metabolic networks. Ultimately, such work could open the way for substantial improvements in organism modifications that could enhance bioleaching performance.

## Author Contributions

K.R.L., S.E.J., T.H.O., B.C.N., K.A.H.‐E., J.F.B. and J.M.S. contributed to conceptualisation and investigation. K.R.L., S.E.J., T.H.O., J.F.B. and J.M.S. contributed to methodology and writing – original draft. K.R.L., S.E.J., T.H.O., L.X.C., K.A.H.‐E., D.G.‐M., J.F.B. and J.M.S. contributed to writing – review and editing. K.R.L., J.F.B. and J.M.S. performed formal analysis. K.R.L., B.C.T. and J.F.B. performed data curation. K.R.L., D.G.‐M., J.F.B. and J.M.S. contributed to visualisation. J.F.B. and J.M.S. provided supervision and, together with K.A.H.‐E., secured funding.

## Funding

This work was supported by the Natural Environment Research Council (NE/L002485/1), Biotechnology and Biological Sciences Research Council (BB/N012674/1) and Hellenic Coppers Mines Ltd.

## Conflicts of Interest

The authors declare no conflicts of interest.

## Supporting information


**Figure S1:** Phylogenetic trees. The phylogenetic trees of Archaea (A) and Bacteria (B) show the evolutionary relationships between microorganisms isolated from the SC3 metagenomes (blue) and references from NCBI (black). Reference NCBI GenBank accession numbers are included in the tree after reference taxonomy. GC Skew of Plasmid_Leptospirillum_56_1137.

**Figure S2:** Plasmid replication types. Overall, across the plasmids the observed GC skew was low and the patterns were noisy, but some of the three plasmids' skew might be consistent with a rolling circle, for example plasmid Plasmid_Leptospirillum_56_137 shown here.

**Table S1:** Metagenome sample data. Coverage of assembly was calculated by mapping reads of the source sample to the assembly.

**Table S2:** Coverage of organisms in each sample, Figure 2A. Coverage of genomes was calculated by mapping reads of the source sample to the genome.

**Table S3:** Chemical analysis of incubations.

## Data Availability

Read data and draft genomes are available on NCBI PRJNA 1170356. Draft genomes are also available on https://ggkbase.berkeley.edu/cu_bioleaching_organisms_and_plasmids. NCBI accession information for genomes is also listed in Table S2.
