# Supplementary figures and images for: Bioleaching Microbial Community Metabolism and Composition Driven by Copper Sulphide Mineral Type
Source: Environ Microbiol Rep. 2025 Dec 11;17(6):e70261. doi: 10.1111/1758-2229.70261 (PMC12698208; doi:10.1111/1758-2229.70261)

# JSantini\_SS8\_scaffold\_33 GC Skew

(window = 1000, slide = 10)

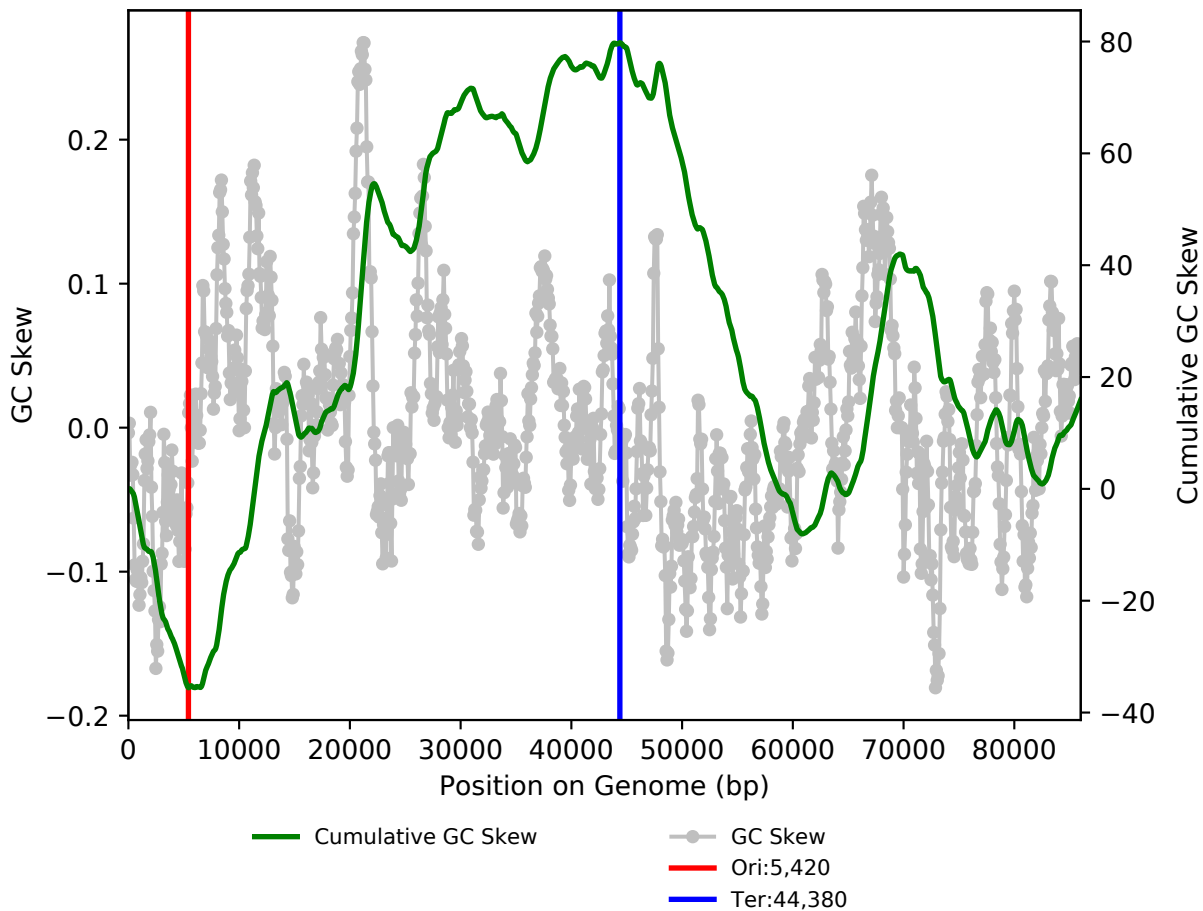

Supplement: Supplementary file 2 — Figure S2: Plasmid replication types. Overall, across the plasmids the observed GC skew was low and the patterns were noisy, but some of the three plasmids' skew might be consistent with a rolling circle, for example plasmid Plasmid_Leptospirillum_56_137 shown here. [file EMI4-17-e70261-s002.pdf]
